# Zenker’s diverticulum perforation due to transoesophageal echocardiography: Case report of the management of an extremely rare life-threatening iatrogenic complication

**DOI:** 10.1016/j.ijscr.2020.06.016

**Published:** 2020-06-11

**Authors:** F.-X. Terryn, P. Stangherlin, B. Mansvelt

**Affiliations:** Department of General Surgery, Hôpital de Jolimont, Haine-Saint-Paul, Belgium

**Keywords:** Zenker’s diverticulum, Transoesophageal echocardiography, Perforation, Case report

## Abstract

**Introduction:**

Transoesophageal echocardiography is generally considered to be a safe monitoring and diagnostic tool. Even though the severe morbidity rate is relatively low and mortality rate exceptional, sometimes life-threatening events may occur during this procedure.

**Presentation of case:**

We described the case of a 79-year-old woman with an extremely rare case of Zenker’s diverticulum perforation following a transoesophageal echocardiography and its successful management with a 6 months follow-up.

**Discussion:**

Several specific endoscopic techniques have been described in the literature in case of Zenker’s diverticulum and we believe the use of check lists should be implemented.

**Conclusion:**

We hope that the documentation of this rare iatrogenic complication will remind the operators of this procedure to be aware of a documented Zenker’s diverticulum and when necessary, to take the published precautions to prevent a highly severe complication.

## Highlights

• Transoesophageal echocardiography has a low severe morbidity rate and an exceptional mortality rate.
• Zenker’s Divertculum perforation is a life-threatening complication which may occur during this procedure and may require emergency surgery.
• Some techniques are described in the literature to perform a safe transoesophageal echocardiography in patients with Zenker’s diverticulum when necessary.
• Check lists should be implemented in order to avoid foreseeable complications.

## 1 Introduction

Transoesophageal echocardiography is commonly considered to be a safe monitoring and diagnostic tool. Its use has significantly increased over the past decades since the first work published by Frazin in 1976 [1]. Even though the severe morbidity rate is relatively low and mortality rate exceptional, sometimes life-threatening events may occur during this procedure. The aim of this article is to document an extremely rare case of Zenker’s diverticulum perforation related to transoesophageal echocardiography and its successful management.

This work has been reported in line with the SCARE criteria [2].

## 2 Presentation of case

A 79-year-old woman presented to the emergency department of another institution with hemianopsia. The diagnosis of occipital cerebrovascular accident was confirmed. An etiologic assessment was performed, including a transoesophageal echocardiography. A Zenker’s diverticulum was mentionned in her medical history amongst other less case-relevant conditions.

**Fig. 1:**
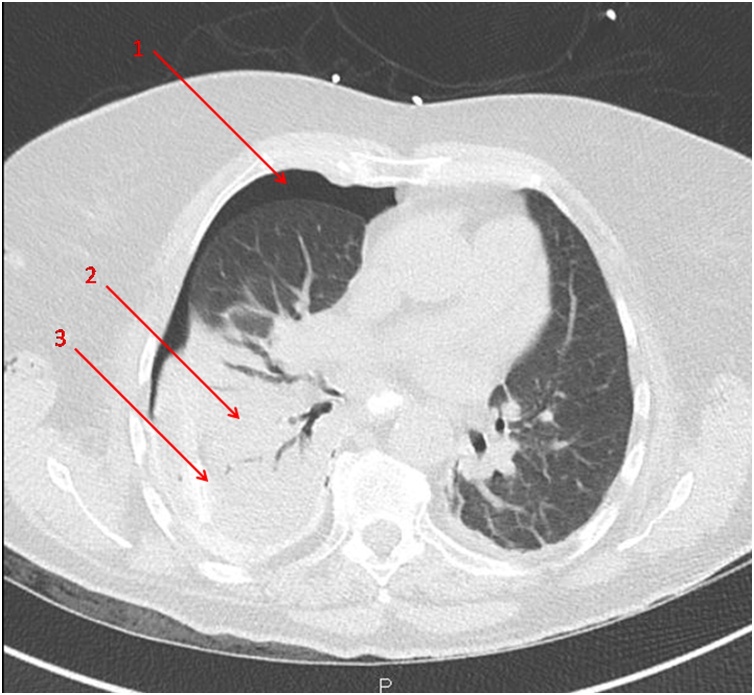
Preoperative CT scan. 1 : pneumothorax 2 : atelectasia 3 : pleural effusion.

In the moments following the procedure, the patient complained of dorsal pain and dyspnea. The clinical examination revealed a cervical subcutaneous emphysema. A CT scan showed a right hydropneumothorax and hydromediastinum (Fig. 1).

At first, a right thoracic drainage was performed and helped stabilising the patient’s condition.

**Fig. 2:**
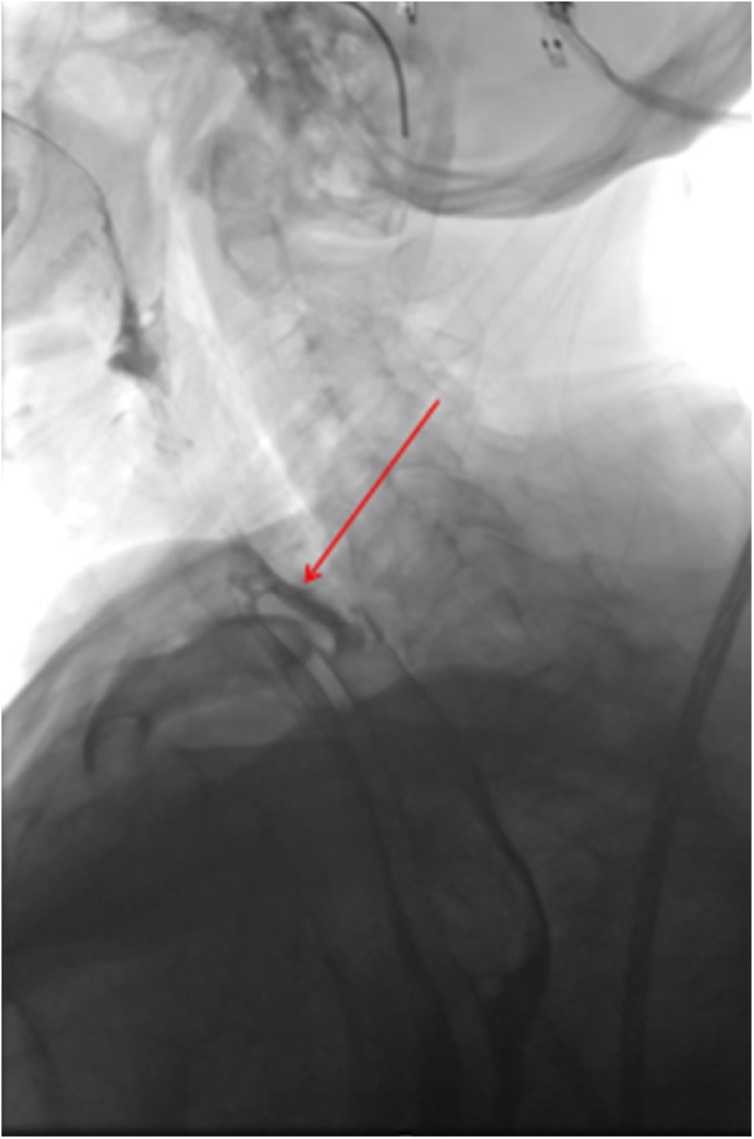
Upper GI study : the red arrows indicated the origin of the Zenker’s diverticulum perforation and opacification reveals a large retro-oesophageal cavity.

An oesophageal tear was almost certain. An upper GI study was nevertheless performed and allowed to confirm the hypothesis of a Zenker’s diverticulum perforation (Fig. 2).

The patient was then transferred to our institution for oesophageal surgery.

**Fig. 3:**
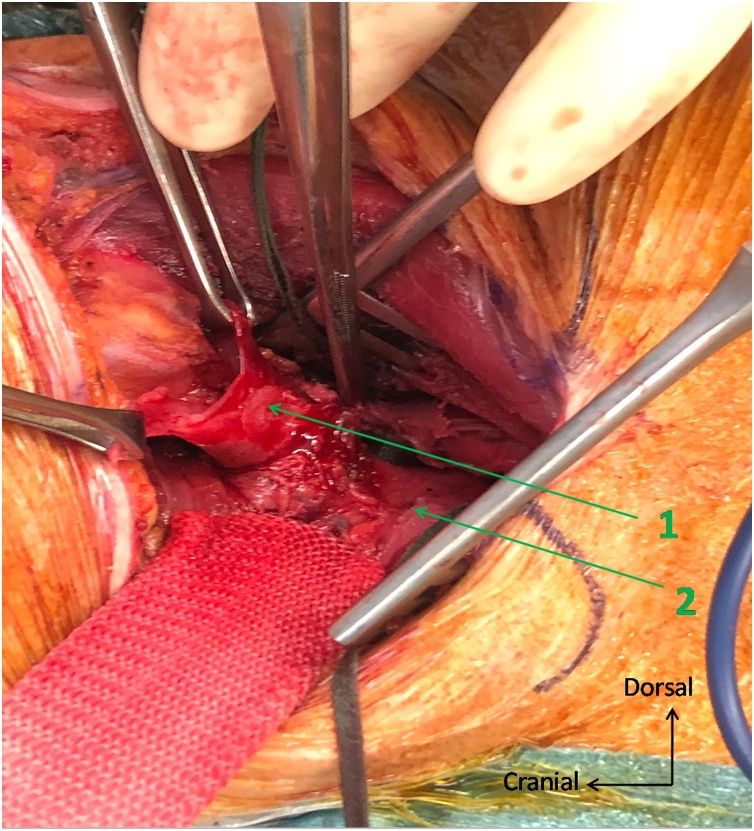
Peroperative photograph : the site of perforation of the Zenker’s diverticulum is identified with the help of a nasogastric tube (not shown on this picture) and complete dissection of the diverticulum is performed before stapling. 1 : perforated diverticulum 2 : cervical oesophagus.

The exploration by a left cervicotomy was performed 26 h after the start of symptoms and confirmed a large perforation at the bottom of a Zenker's diverticulum, with a severe narrowing (8 mm in diameter) of the normal oesophageal route at the level of the cricopharyngeal muscle. We also observed a large (1 cm in diameter) cylindric channel between the oesophagus and the thoracic spine, which was the fistulous tract between the perforation and the right pleura. From these observations, we believe that the transoesophageal echocardiography had been performed with the instrument in this traumatic tract. We performed a resection of the Zenker’s diverticulum by stapling, a cricomyotomy and a mediastinal drainage by left cervicotomy (Fig. 3).

Despite the several complications described below, the postoperative outcome was favourable.

**Fig. 4:**
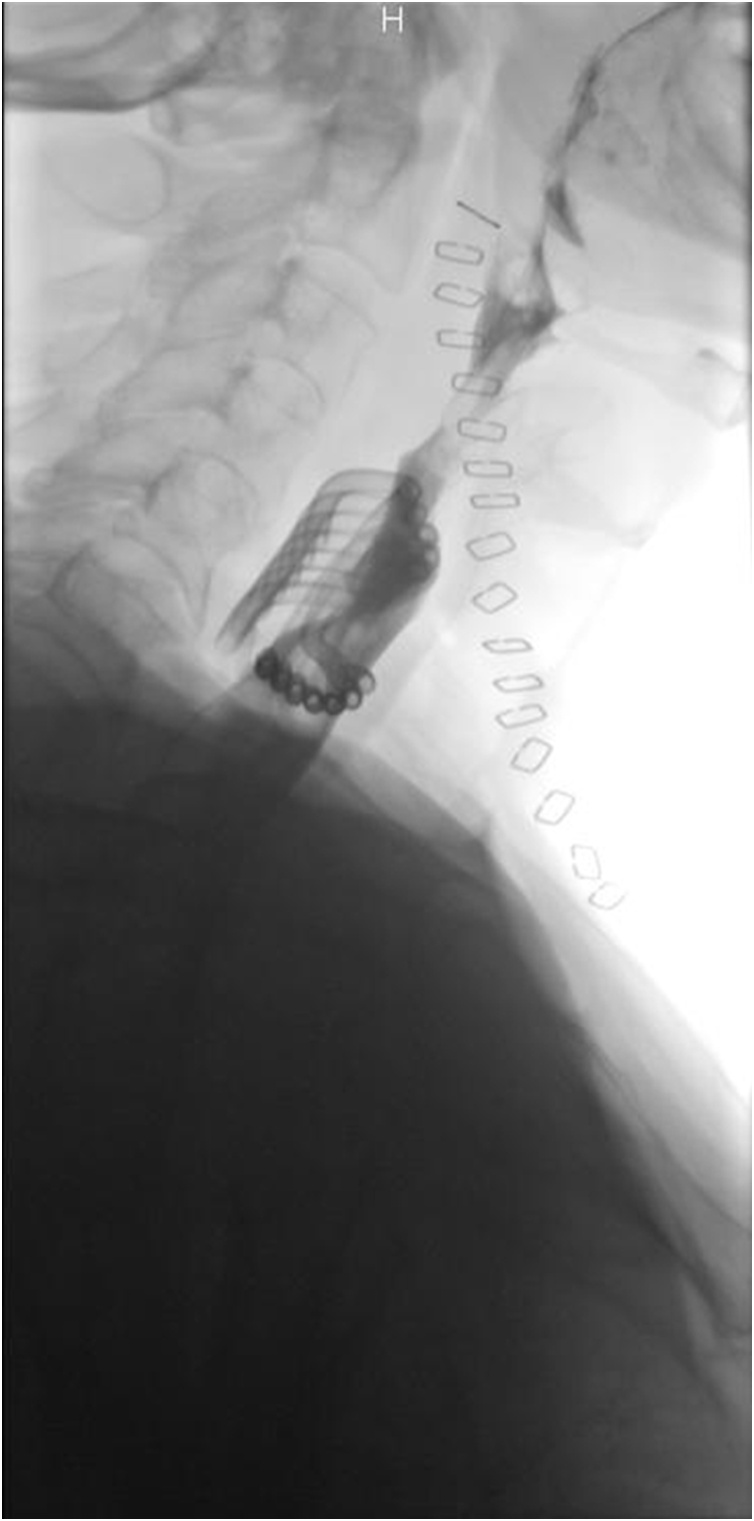
Postoperative upper GI study : no leak or stenosis; multitubular drainage in front of the suture.

Postoperative per os methylene blue test and upper GI series did not reveal any sign of leak or stenosis (Fig. 4).

The patient developed a right-sided pleural abcess requiring appropriate antibiotherapy and an iterative thoracic drainage. Daily chest physiotherapy was also prescribed.

In relation to the mediastinitis, the patient suffered from a paroxystic atrial fibrillation with rapid ventricular response, successfully treated with Amiodarone and beta-blockers.

In order to prevent an inhalation pneumonia, in the context of a recent stroke, a temporary parenteral nutrition was initiated, likely responsible for a de novo type 2 diabetes mellitus onset, requiring insuline therapy. In total, the patient stayed four days in the ICU, eleven days in our surgical station and was allowed to go home after a 27-day complementary stay in the geriatric department (Dindo-Cavien IVb).

At the six months follow-up, the patient was in good physical health and free of symptoms.

## 3 Discussion

We believe that the patient or the medical staff should always notify the existence of a diverticulum of the cervical oesophagus to the performer of a transoesophageal echocardiography if not clearly mentionned in the medical history and it should always be excluded when the operator feels a resistance during the exam. The awareness of this pathology is essential to prevent a dramatic complication such as a diverticulum perforation.

Zenker’s diverticulum results from a dorsal herniation of oesophageal mucosa into the zone of least resistance located above the cricopharyngeus muscle and below the inferior pharyngeal constrictor muscle, called Killian's triangle. Most common symptoms are dysphagia, halitosis, chronic coughing and regurgitation sometimes leading to aspiration pneumonia. Patients with Zenker’s diverticulum have an increased susceptibility to develop an epidermoïd cancer. Its prevalence is around 0,2–1% and its incidence rate approximatively 2/100 000/year. The sex ratio is around 3 men for one women and the age of incidence is generally above 70 years [3].

Another type of cervical oesophagus herniation exists, called Killian-Jamieson diverticulum. It is 4 times less frequent than Zenker’s Diverticulum, and is located below the cricopharyngeal muscle, anterolaterally, around C5-C6 and mostly asymptomatic [4].

Côté and Denault [5] made a review of literature about every publication from 1975 to 2008 mentioning transoesophageal echocardiography related complications. According to this study, only 3 cases of Zenker’s diverticulum’ perforation due to endoscopic echocardiography were described during this period. To our knowledge, no other case has been documented in the more recent literature.

Numerous types of severe procedure complications were described in the literature, such as aspiration pneumonia, gastrointestinal bleeding, arrythmia, larynx and airway trauma and, of course, perforation [6].

Perforation and bleeding rates of 0.01% and 0.03% respectively were recorded in a large cohort of 7200 patients who underwent transoesophageal echocardiography during cardiac surgery [7]. Oesophageal perforation results in longer hospital stays and 20–30% mortality rates [8]. Signs and symptoms such as subcutaneous emphysema, chest or dorsal pain and dyspnea are noticed faster if the patient undergoes the procedure without general anaesthesia. If not diagnosed and rapidly treated, it can lead to mediastinis, sepsis, multiorgan failure and death. Diagnostic imaging generally include upper GI series, chest radiographs or, even better, chest CT scans.

The authors mentionned above also suggested an interesting example of a check list in order to reduce the risk of occurrence of potentially life-threatening and predictable complications, based on the patient’s history and clinical examination [5]. We also believe that this should be systematically implemented.

Several endoscopic techniques have been described to avoid a perforation during transoesophageal echocardiography in patients with oesophageal diverticulum, including the use of an overtube [9], a fluoroscopic balloon-guided probe [10], or a guide wire and then an overtube [11,12]. These can be useful as the transoesophageal echocardiography can sometimes be crucial to determine the etiology of a pathology in these patients.

Regarding the surgical technique, we decided to perform an open surgery and diverticulum stapling but a hand-sewn diverticulectomy could have been a good alternative. In this case of perforation, a diverticulopexy, a diverticulum invagination or even an endoscopic approach were of course no viable options [3].

## 4 Conclusion

We hope that the documentation of this rare iatrogenic complication will remind the operators of this procedure to be aware of a documented Zenker’s diverticulum and when necessary, to take the published precautions to prevent a highly severe complication.

Written informed consent was obtained from the patient for publication of this case report and accompanying images. A copy of the written consent is available for review by the Editor-in-Chief of this journal on request.

## Sources of funding

All authors declare to have no source of fundings.

## Ethical approval

The study is exempt from ethnical approval in our institution.

## Consent

We obtained the patient’s consent and added a consent section in the manuscript.

## Author contribution

All authors were in charge of the patient and contributed equally in the realisation of this manuscript.

Pierre Stangherlin is the surgeon who performed the operation.

I (François-Xavier Terryn) am the trainee surgeon and assisted the intervention.

Baudouin Mansvelt is the head of our surgical department.

We all took care of the patient during her stay.

I was in charge of the redaction of the case and both of the co-authors made interesting comments on my first version of this case report, especially about the discussion and helped with the review of the literature.

## Registration of research studies

Not applicable for this case report.

## Guarantor

François-Xavier Terryn.

## Provenance and peer review

Not commissioned, externally peer-reviewed.

## Declaration of Competing Interest

All authors declare to have no conflicts of interest.
